# Subjective Well-Being of Chinese Sina Weibo Users in Residential Lockdown During the COVID-19 Pandemic: Machine Learning Analysis

**DOI:** 10.2196/24775

**Published:** 2020-12-17

**Authors:** Yilin Wang, Peijing Wu, Xiaoqian Liu, Sijia Li, Tingshao Zhu, Nan Zhao

**Affiliations:** 1 Key Laboratory of Behavioral Science Institute of Psychology Chinese Academy of Sciences Beijing China; 2 Department of Psychology University of Chinese Academy of Sciences Beijing China; 3 Department of Psychology Nankai University Tianjin China; 4 Beijing University of Posts and Telecommunications Beijing China

**Keywords:** COVID-19, residential lockdown, subjective well-being, online ecological recognition

## Abstract

**Background:**

During the COVID-19 pandemic, residential lockdowns were implemented in numerous cities in China to contain the rapid spread of the disease. Although these stringent regulations effectively slowed the spread of COVID-19, they may have posed challenges to the well-being of residents.

**Objective:**

This study aims to explore the effects of residential lockdown on the subjective well-being (SWB) of individuals in China during the COVID-19 pandemic.

**Methods:**

The sample consisted of 1790 Sina Weibo users who were residents of cities that imposed residential lockdowns, of which 1310 users (73.18%) were female, and 3580 users who were residents of cities that were not locked down (gender-matched with the 1790 lockdown residents). In both the lockdown and nonlockdown groups, we calculated SWB indicators during the 2 weeks before and after the enforcement date of the residential lockdown using individuals’ original posts on Sina Weibo. SWB was calculated via online ecological recognition, which is based on established machine learning predictive models.

**Results:**

The interactions of time (before the residential lockdown or after the residential lockdown) × area (lockdown or nonlockdown) in the integral analysis (N=5370) showed that after the residential lockdown, compared with the nonlockdown group, the lockdown group scored lower in some negative SWB indicators, including somatization (*F*_1,5368_=13.593, *P*<.001) and paranoid ideation (*F*_1,5368_=14.333, *P*<.001). The interactions of time (before the residential lockdown or after the residential lockdown) × area (developed or underdeveloped) in the comparison of residential lockdown areas with different levels of economic development (N=1790) indicated that the SWB of residents in underdeveloped areas showed no significant change after the residential lockdown (*P*>.05), while that of residents in developed areas changed.

**Conclusions:**

These findings increase our understanding of the psychological impact and cost of residential lockdown during an epidemic. The more negative changes in the SWB of residents in developed areas imply a greater need for psychological intervention under residential lockdown in such areas.

## Introduction

Since the outbreak of COVID-19, therapeutic options to treat the disease have been limited, although research to produce an effective and safe vaccine is quickly progressing. Varying degrees of travel limitations on citizens, such as residential lockdowns, were enforced across China to contain the epidemic. Residential lockdown is a type of home quarantine during which residents are not allowed to go out unnecessarily and each family can only appoint a relatively fixed family member to purchase living supplies every 2-3 days. These vigorous actions played a key role in containing the spread of COVID-19 [1,2]; however, they have also had some effects on residents’ mental health, including their subjective well-being (SWB) [3,4]. In psychology, the concepts of well-being, mental health, and happiness are often used as synonyms [5]. The World Health Organization (WHO) defined mental health as a “state of well-being whereby individuals recognize their abilities, are able to cope with the normal stresses of life, work productively and fruitfully, and make a contribution to their communities” [6].

As a comprehensive psychological indicator, SWB describes an individual’s subjective evaluation of their living quality and happiness [7]. It focuses on personal experience in a specific social context to reflect individuals’ mental health [8-11]. As a social group experience [11], SWB has also become a surveying mark of government action, governance capability, and social development trends [12,13]. During the COVID-19 crisis, the loss or significant reduction of SWB may not only increase the incidence of panic disorder, anxiety, depression, and other mental health issues [14] but also cause a series of problems at the social level, such as social hindrance, unstable social environment, and decline of trust in the government [15]. To adequately and effectively prepare for the psychological impact and cost of residential lockdown (eg, targeted psychological crisis intervention), it is necessary to examine the mental health of residents from the SWB perspective.

At present, the assessment of positive SWB indicators mainly relies on self-report inventories such as the Psychological Well-Being (PWB) scale [16]. In addition, because mental health is the crucial element or is even considered to be a synonym of SWB [5,6], some psychiatric status rating scales are widely used to assess negative SWB indicators [17,18], such as the Symptom Checklist 90 (SCL-90), with good reliability and validity [19-21].

However, it is almost impossible to conduct a SWB survey measuring the immediate psychological impact of residential lockdown without delay, as it is difficult to anticipate whether and when a residential lockdown will start, and a questionnaire cannot be used for ecological momentary assessment [22,23]. On the other hand, retrospective measurement is also unsuitable for this aim because individuals’ recall bias may result in inability to accurately trace back to the original baseline state [24]. Facing these challenges in conducting an accurate pre-post comparative analysis during unexpected residential lockdown, it is necessary to develop a new method to capture the SWB status of a large number of individuals in a timely fashion.

With the rise of interdisciplinary research, information technology and computing models are increasingly being used in the evaluation of psychological characteristics, and the popularity of web-based social media provides a new platform for such research. Information collected from these social media platforms can clarify the public’s response and can be mapped to psychological indices [25]. For example, Kosinski et al [26] used digital records on Facebook to automatically and accurately predict users’ personal attributes. Qiu et al [27] also showed that users’ personalities manifested on web-based social media. In China, Sina Weibo is a leading social media platform; by March 2020, it comprised more than 550 million monthly active users. By using machine learning–trained predictive models, users’ ecological behavior on Sina Weibo can be tracked to identify their psychological traits, such as their mental health status [28]. Based on web-based big data, these prediction models provided a rationale for our research. According to previous studies, online ecological recognition (OER) can be defined as the method of acquiring users’ psychological characteristics by machine learning models based on ecological web-based social media data [29,30]. Therefore, in this study, to effectively examine the impact of the residential lockdown on SWB, we conducted OER on publicly available Sina Weibo data to identify users’ SWB with high ecological validity on a large scale.

In the present study, we aimed to explore the impact of the residential lockdown on SWB of individuals during the COVID-19 pandemic. To examine individuals’ SWB before and after the residential lockdown, we used data from posts by active Sina Weibo users to calculate SWB indicators through OER based on trained predictive models [5]. The main purpose of this study was to obtain empirical evidence to provide deep insight into the psychological impact and cost of the residential lockdown. This will in turn form the basis for a concrete plan to develop and implement targeted intervention policies to cope with the COVID-19 pandemic efficiently and effectively.

## Methods

### Sampling

We searched for the following keywords on Baidu (baidu.com): *residential lockdown* (居民出行管控) + *city* or *211 measure* (211举措; the abbreviation of the residential lockdown policy) + *city*. After locating the cities that had issued residential lockdowns from January 20 to February 20, 2020, we finally obtained 17 prefecture-level cities in which local governments implemented the same residential lockdown policy. Then, we identified the specific time period for the implementation of the residential lockdown from the announcement released by the local COVID-19 Prevention and Control Headquarters, and we took the enforcement date as the demarcation point of this study. The residential lockdown cities and the enforcement and ending dates of their residential lockdowns are shown in Table 1. Given that the lockdown enforcement durations of Ningbo, Taizhou, and Yiwu were all <2 weeks, we excluded these three residential lockdown cities from the subsequent analysis.

**Table 1:** Residential lockdown cities and their enforcement and ending dates (in 2020).

Residential lockdown city	Province	Enforcement date	Ending date
Bengbu	Anhui	February 3	March 22
Ezhou	Hubei	February 4	March 25
Fangchenggang	Guangxi Zhuang Autonomous Region	February 2	March 6
Fuyang	Anhui	February 5	March 9
Guigang	Guangxi Zhuang Autonomous Region	February 3	February 24
Huaibei	Anhui	February 3	March 20
Huanggang	Hubei	February 1	March 22
Huangshi	Hubei	February 17	March 23
Nanyang	Henan	February 4	March 11
Ningbo^a^	Zhejiang	February 4	February 10
Songyuan	Jilin	February 4	March 20
Taizhou^a^	Zhejiang	February 2	February 13
Wenzhou	Zhejiang	February 1	February 19
Wuhan	Hubei	February 11	April 8
Xiaogan	Hubei	February 14	March 14
Yiwu^a^	Zhejiang	February 4	February 12
Zhongshan	Guangdong	February 7	March 27

^q^Ningbo, Taizhou, and Yiwu were not included in subsequent analyses because the enforcement duration in these cities was less than 2 weeks.

### Participants and Data Collection

The sample in this study was obtained from the original Sina Weibo data pool [30] containing more than 1.16 million active users. The retrieved data included users’ account profiles and posts. Privacy was strictly protected during this process, referring to the ethical principles listed by Kosinski et al [31]. This study was approved by the Review Board of Institute of Psychology, Chinese Academy of Sciences (ethical code H15009).

From the data pool, we selected Sina Weibo users who met the following criteria: (1) Published at least one original Weibo post per day on average within 2 weeks before and after the corresponding enforcement date of the residential lockdown (see Table 2); (2) authentication type was no-institutional, such as individual user; (3) the geolocation of the user’s account profile was in China, not “overseas” or “others.”

#### Lockdown Group

We combined all the residential lockdown cities mentioned in Table 1 except Ningbo, Taizhou, and Yiwu as the lockdown group. Then, we downloaded each user’s original posts during the 2 weeks before and after the enforcement date of the residential lockdown in their city of residence for analysis in this study.

#### Nonlockdown Group

We combined users who did not live in the abovementioned 17 residential lockdown cities as the entire nonlockdown sample group. Because cities under residential lockdown are a minority among all the cities in China, to increase the generalizability of the results and reduce sampling error, we built the nonlockdown group at twice the sample size of the lockdown group in each of the 14 residential lockdown cities by random sampling without replacement of users in the whole nonlockdown sample group. The male-female ratio of the nonlockdown group was same as that of the lockdown group. Finally, we downloaded each user’s original posts during the 2 weeks before and after the enforcement date from the paired residential lockdown city for analysis in this study.

The final sample contained 5370 users, among whom the male-female ratio was 1:2.7 (see Table 2 for details).

**Table 2:** Numbers of users in each group in the final sample and corresponding time periods of data extraction (N=5370).

Residential lockdown city	Time period (in 2020)		Lockdown group	Nonlockdown group
	Before the residential lockdown	After the residential lockdown		
Bengbu	January 21 to February 3	February 4-17	25	50
Ezhou	January 21 to February 3	February 4-17	16	32
Fangchenggang	January 19 to February 1	February 2-15	18	36
Fuyang	January 22 to February 4	February 5-18	14	28
Guigang	January 20 to February 2	February 3-16	32	64
Huaibei	January 20 to February 2	February 3-16	25	50
Huanggang	January 18-31	February 1-14	28	56
Huangshi	February 3-16	February 17 to March 1	11	22
Nanyang	January 21 to February 3	February 4-17	42	84
Songyuan	January 21 to February 3	February 4-17	16	32
Wenzhou	January 19 to February 1	February 2-15	235	470
Wuhan	January 28 to February 10	February 11-24	1084	2168
Xiaogan	January 31 to February 13	February 14-27	10	20
Zhongshan	January 24 to February 6	February 7-20	234	468

### Compartmentalization by Economic Development Level

SWB is influenced by various factors in the long term [32-35], and there is an inevitable connection between the level of economic development and SWB. Clark and Senik [36] reported that economic development was generally identified with growth in gross domestic product (GDP) per capita, which improved residents’ welfare [37]. Therefore, differentiating the lockdown group by GDP per capita could clarify the impact of the residential lockdown under different economic development conditions.

We took the GDP per capita published by the Municipal Bureau of Statistics of each residential lockdown city in 2017 as the indicator of economic development level. Using between-groups linkage and taking the squared Euclidean distance as the measurement standard, we applied a hierarchical clustering analysis on the 14 residential lockdown cities included in our study. The dendrogram of the whole process of clustering indicated that when the 14 residential lockdown cities were divided into 2 categories, the distance between the categories was relatively large and the characteristics of each category were relatively prominent; therefore, the categories could be readily defined. We applied an additional hierarchical clustering analysis with a cluster number of 2 on the 14 residential lockdown cities. The results suggested that the first category included Wuhan, Ezhou, Zhongshan, and Fangchenggang, and the second category included the remaining 10 residential lockdown cities. Based on this result, in this study, we grouped Wuhan, Ezhou, Zhongshan, and Fangchenggang into developed areas with residential lockdowns and grouped the remaining 10 residential lockdown cities into underdeveloped areas with residential lockdowns. All analyses were performed using SPSS Statistics 26 (IBM Corporation).

### Measurement of SWB and Procedure

We applied OER by employing established machine learning models [28,38] to calculate the SWB indicators of the users in the final sample before and after the corresponding enforcement date of the residential lockdown. First, we used the TextMind system [39], a Chinese language psychological analysis system, to extract psycholinguistic features from active Sina Weibo users’ original posts. This system first segmented the users’ original Weibo posts and extracted independent and linguistically labeled words [40]; then, it used the Simplified Chinese Language Inquiry and Word Count (SCLIWC) to determine the word frequency statistics [41].

Next, we used these extracted psycholinguistic features as the input of established machine learning models to calculate the SWB indicators. The SWB indicators in our research included both positive and negative indicators, which are depicted in Table 3. Because the SCL-90 is also widely used in the assessment of negative SWB indicators [17], we used factors of the SCL-90 as our negative SWB indicators.

**Table 3:** Positive and negative SWB indicators.

SWB^a^ indicator		Definition
**Positive^b^**		
	Self-acceptance	Possession of a positive attitude toward the self
	Environmental mastery	Sense of mastery and competence in managing the environment
	Positive relations	Warm, satisfying, trusting relationships with others
	Purpose in life	Goals in life and a sense of directedness
	Personal growth	Feeling of continued development
	Autonomy	Self-determining and independent
**Negative^c^**		
	Somatization	Distress arising from perceptions of bodily disfunction
	Obsessive-compulsive	Thoughts, impulses, and actions that are experienced as unremitting and irresistible by the individual, but are of an ego-alien or unwanted nature
	Interpersonal sensitivity	Feelings of personal inadequacy and inferiority, particularly in comparison to other persons
	Depression	Withdrawal of life interest, lack of motivation, and loss of vital energy
	Anxiety	Restlessness, nervousness, and tension
	Hostility	Thoughts, feelings, or actions that are characteristics of the negative affect state of anger
	Phobic anxiety	Persistent fear response to a specific person, place, object, or situation which is characterized as being irrational and disproportionate to the stimulus, and which leads to avoidance or escape behavior
	Paranoid Ideation	Paranoid behavior fundamentally as a disordered mode of thinking
	Psychoticism	Withdrawn, isolated, schizoid lifestyle, and first-rank symptoms of schizophrenia.

^a^SWB: subjective well-being.

^b^Source: Psychological Well-Being scale [42].

^c^Source: Symptom Checklist 90 [19].

We used Li, Hao, Bai, and Zhu’s machine learning model [28] to calculate the positive SWB indicators. To establish this predictive model, 1785 active Sina Weibo users were invited to complete Scales of PWB on the internet [42] and were asked for approval to access their Weibo data. Then, we used multivariate adaptive regression splines and other algorithms to fit the relationship between the psycholinguistic features and self-report scores, and the evaluation was conducted by 10-fold cross-validation. To predict the negative SWB indicators, we used Hao et al’s [38] machine-learning model to predict them. To establish this model, we used Weibo data of 448 active Sina Weibo users and their SCL-90 scores [19]. We used a linear regression algorithm to fit the relationship between the psycholinguistic features and self-report scores and conducted 10-fold cross-validation. For most SWB indicators, the Pearson correlation coefficient between the predicted and self-report scores achieved a moderate level (≥.30) [28,38]. For more details about the established SWB models, please see [28,38].

To explore the overall impact of the residential lockdown on SWB, we conducted 2 (times: before the residential lockdown, after the residential lockdown) × 2 (areas: lockdown, nonlockdown) repeated measures analysis of variance (RM ANOVA) on all our samples. In this analysis, time was the within-subject factor, while area was the between-subject factor. Moreover, to explore the impact of the residential lockdown on the SWB of residents at different economic development levels, we applied 2 (times: before the residential lockdown, after the residential lockdown) × 2 (areas: developed, underdeveloped) RM ANOVA on the residential lockdown cities. All these analyses were performed using SPSS 26.

## Results

### Demographics

Among the 5370 active Sina Weibo users, 1790 were in the lockdown group (1310 female, 73.18%) and 3580 were in the non-lockdown group (gender matched with the lockdown group). According to the birthdates the users registered in their account profiles (1160/5370, 21.60%), the users’ ages ranged from 21 to 83 years, with a median age of 29.95 (SD 6.30) years. Table 4 features the demographic profiles of these users.

**Table 4:** Demographic characteristics of users in the lockdown group and nonlockdown group (N=5370), n (%).

Characteristic		Developed areas with residential lockdown (n=1352)	Underdeveloped areas with residential lockdown (n=438)	Lockdown group (n*=*1790)	Nonlockdown group (n*=*3580)
**Gender**					
	Male	348 (25.74)	132 (30.14)	480 (26.82)	960 (26.82)
	Female	1004 (74.26)	306 (69.86)	1310 (73.18)	2620 (73.18)
**Age (years)**					
	18-30	198 (14.64)	57 (13.01)	255 (14.25)	464 (12.96)
	31-40	92 (6.80)	36 (8.22)	128 (7.15)	249 (6.96)
	≥41	11 (0.81)	7 (1.60)	18 (1.01)	46 (1.28)
Missing data		1051 (77.74)	338 (77.17)	1389 (77.60)	2821 (78.80)

### All Residential Lockdown Cities

In this analysis, we compared the SWB scores of residents of different areas across the country during different periods. As shown in Table 5, there were significant (including marginally significant) interactions of area and time on 2 negative SWB indicators.

**Table 5:** Repeated measures analysis of variance on subjective well-being across China.

Indicator	Residential lockdown area, mean (SD)				Area		Time		Area × Time	
	Lockdown group (n=1790)		Nonlockdown group (n=3580)		*F_1,5368_*	*P* value	*F_1,5368_*	*P* value	*F_1,5368_*	*P* value
	T-before^a^	T-after^b^	T-before	T-after						
Somatization	8.38 (2.94)	8.64 (3.04)	8.56 (3.25)	8.98 (3.30)	10.457	.001	56.414	<.001	3.430	.06
Paranoid Ideation	5.46 (1.46)	5.61 (1.45)	5.58 (1.85)	5.82 (2.11)	12.418	<.001	66.862	<.001	3.405	.07

^a^T-before: the period before the residential lockdown.

^b^T-after: the period after the residential lockdown.

#### Somatization

The main effect of the area was significant (*F*_1,5368_=10.457, *P*=.001, η_p_^2^=0.002). The main effect of time was significant (*F*_1,5368_=56.414, *P*<.001, η_p_^2^=0.010. The interaction of area and time was marginally significant (*F*_1,5368_=3.430, *P*=.06, η_p_^2^=0.001. After simple effect analysis, our findings were as follows:

Area: In the T-before period, there was no significant difference between the lockdown group and the nonlockdown group; in the T-after period, the somatization of residents in the lockdown group was significantly lower than that of residents in the non-lockdown group (*F*_1,5368_=13.593, *P*<.001, η_p_^2^=0.003).

Time: After the implementation of the residential lockdown, somatization of the residents in both groups increased significantly (lockdown group: *F*_1,5368_=12.009, *P*=.001, η_p_^2^=0.002; nonlockdown group: *F*_1,5368_=65.748, *P*<.001, η_p_^2^=0.012).

#### Paranoid Ideation

The main effect of area was significant (*F*_1,5368_=12.418, *P*<.001, η_p_^2^*=*0.002). The main effect of time was significant (*F*_1,5368_=66.862, *P*<.001, η_p_^2^=0.012). The interaction of area and time was marginally significant (*F*_1,5368_=3.405, *P*=.07, η_p_^2^=0.001). After simple effect analysis, our findings were as follows:

Area: In both periods, the paranoid ideation of residents in the lockdown group was significantly lower than that of residents in the nonlockdown group (T-before: *F*_1,5368_=6.008, *P*=.01, η_p_^2^=0.001; T-after: *F*_1,5368_=14.333, *P*<.001, η_p_^2^=0.003).

Time: After the implementation of the residential lockdown, the paranoid ideation of residents in both groups increased significantly (lockdown group: *F*_1,5368_=15.033, *P*<.001, η_p_^2^=0.003; non-lockdown group: *F*_1,5368_=75.334, *P*<.001, η_p_^2^=0.014).

However, for self-acceptance, environmental mastery, positive relations, purpose in life, personal growth, autonomy, obsessive-compulsive, interpersonal sensitivity, depression, anxiety, hostility, phobic anxiety, and psychoticism, the interactions of area and time were not significant (all *P* values >.10).

**Figure 1:**
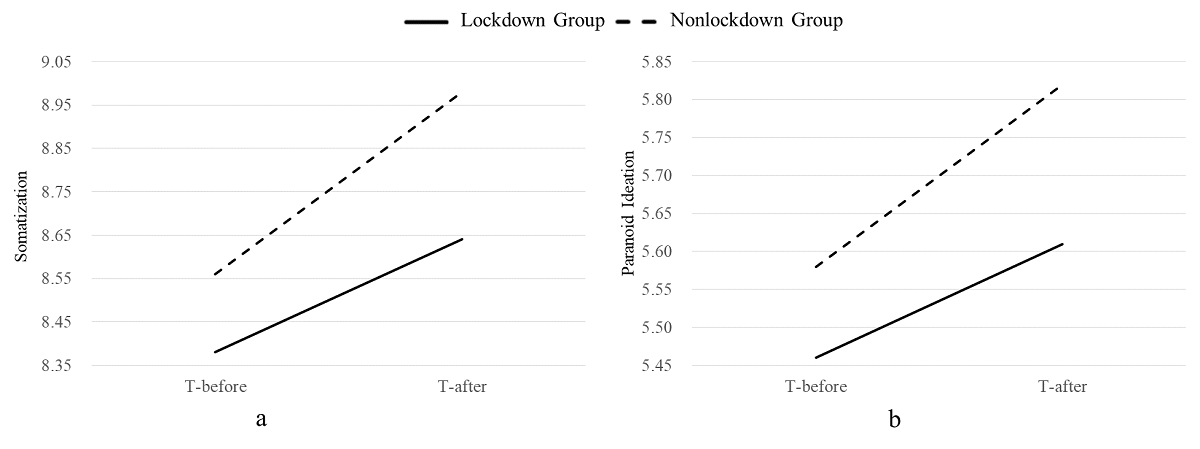
Interactions of area and time on (a) somatization and (b) paranoid ideation across China. T-after: the period after the residential lockdown; T-before: the period before the residential lockdown.

The interactions of somatization and paranoid ideation are presented in Figure 1.

### Residential Lockdown Areas at Different Economic Development Levels

According to the clustering analysis above, we grouped Wuhan, Ezhou, Zhongshan, and Fangchenggang into developed areas with residential lockdowns, and we grouped the remaining 10 residential lockdown cities into underdeveloped areas with residential lockdowns. To further explore the different effects of residential lockdown on the SWB of residents of areas with different economic development, we compared the SWB scores of residents of developed areas and underdeveloped areas during the different periods.

As shown in Table 6, there were significant (including marginally significant) interactions of area and time on 2 positive SWB indicators and 5 negative SWB indicators.

**Table 6:** Repeated measures analysis of variance on subjective well-being in the residential lockdown areas.

Indicator	Residential lockdown area, mean (SD)				Area		Time		Area × Time	
	Developed (n=1352)		Underdeveloped (n=438)		*F_1,1788_*	*P* value	*F_1,1788_*	*P* value	*F_1,1788_*	*P* value
	T-before^a^	T-after^b^	T-before	T-after						
Purpose in life	12.19 (1.23)	12.14 (1.30)	11.96 (1.20)	12.08 (1.53)	5.548	.02	0.698	.40	5.057	.03
Autonomy	10.92 (1.81)	10.84 (1.75)	10.57 (1.85)	10.67 (1.79)	8.351	.004	0.099	.75	4.984	.03
Interpersonal sensitivity	9.31 (2.73)	9.55 (2.74)	9.81 (2.97)	9.63 (2.96)	4.342	.04	0.248	.62	9.850	.002
Anxiety	9.48±3.85	9.99±4.16	10.00 (3.95)	10.04±4.42	1.880	.17	9.527	.002	7.273	.007
Hostility	5.44 (2.00)	5.60 (2.08)	5.75 (2.22)	5.66 (2.13)	3.382	.07	0.466	.50	5.202	.02
Paranoid ideation	5.43 (1.42)	5.61 (1.45)	5.57 (1.55)	5.61 (1.47)	1.155	.28	7.822	.005	3.083	.08
Psychoticism	8.63 (2.55)	8.84 (2.83)	9.03 (2.89)	8.97 (2.69)	4.251	.04	1.074	.30	3.584	.06

^a^T-before: the period before the residential lockdown.

^b^T-after: the period after the residential lockdown.

#### Purpose in Life

The main effect of area was significant (*F*_1,1788_=5.548, *P*=.02, η_p_^2^=0.003). The main effect of time was not significant (*F*_1,1788_=0.698, *P*=.40, η_p_^2^=0.000). The interaction of area and time was significant (*F*_1,1788_=5.057, *P*=.03, η_p_^2^=0.003). After simple effect analysis, our findings were as follows:

Area: In the T-before period, the purpose in life of residents of developed areas with residential lockdowns was significantly higher than that of residents of underdeveloped areas with residential lockdowns (*F*_1,1788_=11.363, *P*=.001, η_p_^2^=0.006); in the T-after period, there was no significant difference between developed and underdeveloped areas with residential lockdowns.

Time: After the implementation of the residential lockdown, there were no significant changes in either developed or underdeveloped areas with residential lockdowns.

#### Autonomy

The main effect of area was significant (*F*_1,1788_=8.351, *P*=.004, η_p_^2^=0.005). The main effect of time was not significant (*F*_1,1788_=.099, *P*=.75, η_p_^2^=0.000). The interaction of area and time was significant (*F*_1,1788_=4.984, *P*=.03, η_p_^2^=0.003). After simple effect analysis, our findings were as follows:

Area: In the T-before period, the autonomy of residents of developed areas with residential lockdowns was significantly higher than that of residents of under-developed areas with residential lockdowns (*F*_1,1788_=12.229, *P*<.001, η_p_^2^=0.007; in the T-after period, there was no significant difference between developed and underdeveloped areas with residential lockdown.

Time: After the implementation of residential lockdown, there were no significant changes in either developed or underdeveloped areas with residential lockdowns.

#### Interpersonal Sensitivity

The main effect of area was significant (*F*_1,1788_=4.342, *P*=.04, η_p_^2^=0.002). The main effect of time was not significant (*F*_1,1788_=0.248, *P*=.62, η_p_^2^=0.000). The interaction of area and time was significant (*F*_1,1788_= 9.850, *P*=.002, η_p_^2^=0.005). After simple effect analysis, our findings were as follows:

Area: In the T-before period, the interpersonal sensitivity of residents of developed areas with residential lockdowns was significantly lower than that of residents of underdeveloped areas with residential lockdowns (*F*_1,1788_=10.643, *P*=.001, η_p_^2^=0.006); in the T-after period, there was no significant difference between developed and underdeveloped areas with residential lockdowns.

Time: After the implementation of residential lockdown, the interpersonal sensitivity of residents in developed areas with residential lockdowns increased significantly (*F*_1,1788_=13.514, *P*<.001, η_p_^2^=0.008); meanwhile, there was no significant change in underdeveloped areas with residential lockdowns.

#### Anxiety

The main effect of area was not significant (*F*_1,1788_=1.880, *P*=.17, η_p_^2^=0.001). The main effect of time was significant (*F*_1,1788_=9.527, *P*=.002, η_p_^2^=0.005). The interaction of area and time was significant (*F*_1,1788_=7.273, *P*=.007, η_p_^2^=0.004). After simple effect analysis, our findings were as follows:

Area: In the T-before period, the anxiety of residents of developed areas with residential lockdowns was significantly lower than that of residents of underdeveloped areas with residential lockdowns (*F*_1,1788_=5.923, *P*=.02, η_p_^2^=0.003); in the T-after period, there was no significant difference between developed and underdeveloped areas with residential lockdowns.

Time: After the implementation of residential lockdown, the anxiety of residents of developed areas with residential lockdowns increased significantly (*F*_1,1788_=34.173, *P*<.001, η_p_^2^=.019); meanwhile, there was no significant change in underdeveloped areas with residential lockdowns.

#### Hostility

The main effect of area was marginally significant (*F*_1,1788_=3.382, *P*=.07, η_p_^2^=0.002). The main effect of time was not significant (*F*_1,1788_=0.466, *P*=.50, η_p_^2^=0.000). The interaction of area and time was significant (*F*_1,1788_=5.202, *P*=.02, η_p_^2^=0.003). After simple effect analysis, our findings were as follows:

Area: In the T-before period, the hostility of residents of developed areas with residential lockdowns was significantly lower than that of residents of underdeveloped areas with residential lockdowns (*F*_1,1788_=7.487, *P*=.006, η_p_^2^=0.004); in the T-after period, there was no significant difference between developed and underdeveloped areas with residential lockdowns.

Time: After the implementation of residential lockdown, the hostility of residents of developed areas with residential lockdowns increased significantly (*F*_1,1788_=8.974, *P*=.003, η_p_^2^=0.005); meanwhile, there was no significant change in underdeveloped areas with residential lockdowns.

#### Paranoid Ideation

The main effect of area was not significant (*F*_1,1788_=1.155, *P*=.28, η_p_^2^=0.001). The main effect of time was significant (*F*_1,1788_=7.822, *P*=.005, η_p_^2^=0.004). The interaction of area and time was marginally significant (*F*_1,1788_=3.083, *P*=.08, η_p_^2^=0.002). After simple effect analysis, our findings were as follows:

Area: In both periods, there were no significant differences between developed and underdeveloped areas with residential lockdowns.

Time: After the implementation of residential lockdown, the paranoid ideation of residents of developed areas with residential lockdowns increased significantly (*F*_1,1788_=21.177, *P*<.001, η_p_^2^=0.012); meanwhile, there was no significant change in underdeveloped areas with residential lockdowns.

#### Psychoticism

The main effect of area was significant (*F*_1,1788_=4.251, *P*=.04, η_p_^2^=.002). The main effect of time was not significant (*F*_1,1788_=1.074, *P*=.30, η_p_^2^=0.001). The interaction of area and time was marginally significant (*F*_1,1788_=3.584, *P*=.06, η_p_^2^=0.002). After simple effect analysis, our findings were as follows:

Area: In the T-before period, the psychoticism of residents of developed areas with residential lockdowns was significantly lower than that of residents of underdeveloped areas with residential lockdowns (*F*_1,1788_=7.822, *P*=.005, η_p_^2^=0.004); in the T-after period, there was no significant difference between developed and underdeveloped areas with residential lockdowns.

Time: After the implementation of residential lockdown, the psychoticism of residents of developed areas with residential lockdowns increased significantly (*F*_1,1788_=8.768, *P*=.003, η_p_^2^=0.005); meanwhile, there was no significant change in underdeveloped areas with residential lockdowns.

However, for self-acceptance, environmental mastery, positive relations, personal growth, somatization, obsessive-compulsive, depression, and phobic anxiety, the interactions of area and time were not significant (all *P* values >.10).

**Figure 2:**
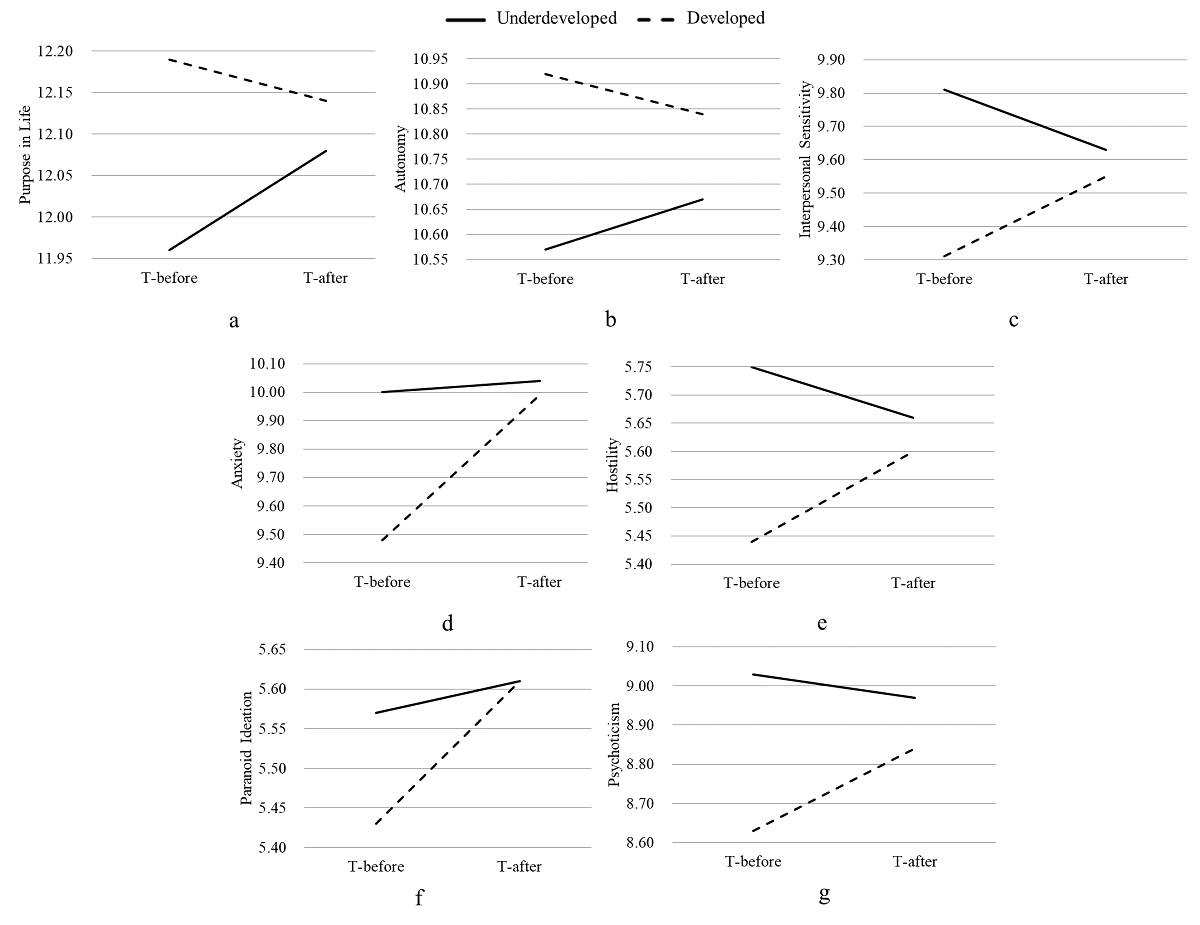
Interactions of area and time on (a) purpose in life, (b) autonomy, (c) interpersonal sensitivity, (d) anxiety, (e) hostility, (f) paranoid ideation, and (g) psychoticism in the residential lockdown areas. T-after: the period after the residential lockdown; T-before: the period before the residential lockdown.

The interactions of purpose in life, autonomy, interpersonal sensitivity, anxiety, hostility, paranoid ideation, and psychoticism are presented in Figure 2.

## Discussion

### Principal Findings

This study aimed to investigate the impact of the residential lockdown on SWB. We obtained the SWB indices of residents inside and outside cities with residential lockdown before and after the enforcement of the residential lockdown policy through OER. In addition, by introducing economic factors, we also made a transverse comparison of SWB in residential lockdown cities divided by economic development level. The results of the RM ANOVAs revealed that the lockdown group scored lower on negative SWB indicators than the nonlockdown group. Moreover, the SWB of residents in underdeveloped areas remained relatively stable after the implementation of the residential lockdown. On the whole, the strict regulations of the residential lockdown and the limitation on residents’ travel did have an impact on SWB; however, this impact was positive, and the degree of positivity was greater in underdeveloped areas.

By comparing the residential lockdown cities with corresponding cities without residential lockdowns all over China, the main effect of time from RM ANOVA indicated that as time progressed, the overall SWB of the residents was adversely affected to some degree by the epidemic. This impact was mainly manifested by a general decrease in the scores of positive SWB indicators and an increase in the scores of negative SWB indicators. These results are consistent with previous studies: COVID-19 has caused public pressure and panic owing to the high degree of uncertainty and infectivity of the disease, which has had a deleterious effect on people’s mental health [4,14].

However, it is worth mentioning that the simple effect of the interaction in the integral analysis indicated that the implementation of the residential lockdown had a positive influence on the control of residents’ negative mental states. Specifically, compared with the nonlockdown group, residents in the lockdown group scored lower on negative SWB indicators. This finding also suggests that stringent residential lockdowns during a pandemic will not only ensure residents’ health and life but will also effectively control the prevailing negative mental states of residents. Our findings are in keeping with those of Suryawanshi and More [43], who reported that the positive impact of the lockdown was far greater than the negative impact. Two factors may explain the above results. First, because the residential lockdowns were implemented only after the outbreak of COVID-19, people had a full understanding of the epidemic and were aware that only residential lockdown could effectively stop the spread of the virus by cutting off the transmission route. Therefore, on balance, because the powerful residential lockdown slowed the spread of the virus and curbed the rapid deterioration of the epidemic, it provided greater psychological comfort to the residents compared with the discomfort caused by restriction of personal freedom [43]. Second, the residential lockdown offered family members an opportunity to reevaluate and improve their family relations [43], which could effectively relieve their state of mind.

Furthermore, we used GDP per capita as an indicator of economic development level to classify the cities under residential lockdown. After performing RM ANOVA, we found that the positive impact of the residential lockdown was different in areas with different economic development levels. The SWB of residents in underdeveloped areas with residential lockdown (eg, Huanggang, Huangshi) was found to be relatively stable over time. In contrast, the results showed that during the lockdown in developed areas (eg, Wuhan, Zhongshan), the SWB of the residents generally changed significantly for the worse, although two SWB indicators did not show significant changes (ie, purpose in life and autonomy). The difference in the stability of residents’ mental states between the residential lockdown areas at high and low economic development levels can mainly be explained by the following reasons.

#### Urban and Rural Population Ratio

Economists generally believe that regional economic growth is related to population structure (eg, urban and rural population ratio), among which GDP per capita is positively associated with urban population size [44]. In the residential lockdown areas with higher economic development, the urban population size is larger, and the problems produced by the residential lockdown (eg, unemployment) had a strong impact on urban commuters, which posed great risks to local residents’ SWB.

#### Communication and Transportation

The statistical yearbooks of the residential lockdown cities reported that in the more economically developed areas, key indicators (eg, passenger traffic, number of civil vehicles) would score higher. For example, in 2017, the passenger traffic of Wuhan was 299.5 million, and the number of civil vehicles was 2.75 million [45]. These figures show that residents of more economically developed areas may have had greater transport demand and therefore were more affected by stringent residential lockdown due to the restrictions on their travel.

#### Floating Population

Economic development is the decisive factor in the formation of population flow. The population usually flows to developed areas with better economic development [46]. During the residential lockdown, the original pattern of life was disrupted, face-to-face association outside home was cut off, and residents mainly obtained social support from family members [47]. However, most members of the floating population make their living without the company of family. Hence, in more economically developed areas, the size of the floating population without family is larger, and the psychological impact of the residential lockdown on residents was more significant.

With the fast rise in the number of confirmed cases, COVID-19 has become a global pandemic. The decision of whether to enforce residential lockdowns is a difficult challenge faced by governments worldwide. The above results indicate that residential lockdowns not only have important medical value in protecting public health and life safety but may also have psychological value in maintaining SWB in China. Furthermore, in developed areas, the urban and floating populations are larger, and the relationship between transportation and life is more important; thus, the SWB of local residents is more likely to be affected by the residential lockdown and fluctuate greatly. These findings provide new references for local governments of areas at different economic development levels to consider the benefit and cost of residential lockdown and to prepare for the possible risks. For example, local governments in developed areas should make more preparations for psychological interventions and associated costs during and following residential lockdown.

To date, Britain, America, and many other countries have adopted lockdowns that are similar to those in China to some extent. Despite cultural differences, the effectiveness of residential lockdown in stopping disease spread is obvious [48]. However, the positive and negative psychological effects of the lockdown are complicated. These effects will also be influenced by the enforcement time, the effects of lockdown, and other factors mentioned above. In addition, in view of the fact that the level of economic development is a common indicator, different regions of the world will also have differences in urban and rural population ratios, floating population, communication and transportation, etc, which may influence the psychological effects caused by residential lockdown. Our findings with regard to the overall effect of the residential lockdown on SWB and the differences in the impact on mental states under different economic development conditions are instructive to analogous policies around the world.

### Limitations

Although this study reveals changes in SWB caused by residential lockdown, there are some limitations to be highlighted. First, considering that most of the respondents in our sample are female, there may be some bias in the results. Second, more than 70% of our sample did not provide age information on the internet, which prevented us from evaluating the possible influence of age. In future studies, obtaining more age information and expanding the ranges of age and gender could be helpful. Third, in this study, transverse comparison of SWB in cities divided by economic development level only targeted residential lockdown areas; therefore, in future studies, this comparison can be extended to areas without residential lockdowns. Fourth, in light of the fact that not only economic factors but other factors such as social environment and cultural background may affect SWB, future studies could consider more factors to obtain a more comprehensive understanding of the lockdown effect. Additionally, it should be noted that the patterns we observed may be largely determined by the Chinese culture, and any application of our conclusions to a different culture should be made very carefully.

### Conclusion

In this study, to explore the impact of residential lockdown during the COVID-19 pandemic on individual SWB in China, we predicted residents’ SWB by OER and compared the SWB of residents of different areas with different economic development levels. The results demonstrated that in China and in the context of the COVID-19 pandemic, the residential lockdown alleviated the negative psychological changes caused by the epidemic to a certain extent. In addition, compared with developed areas, the psychological benefit of the residential lockdown was more obvious in underdeveloped areas. This study sheds light on the psychological effects of strict residential lockdown during pandemics and provides new clues for epidemic prevention in areas with different socioeconomic situations.

## Appendix

Multimedia Appendix 1 CONSORT-eHEALTH checklist (V 1.6.1).

## Abbreviations

GDP: gross domestic product
OER: online ecological recognition
PWB: Psychological Well-Being scale
RM ANOVA: repeated measures analysis of variance
SCL-90: Symptom Checklist 90
SCLIWC: Simplified Chinese Language Inquiry and Word Count
SWB: subjective well-being
WHO: World Health Organization
